# A Multi-Attention Approach for Person Re-Identification Using Deep Learning

**DOI:** 10.3390/s23073678

**Published:** 2023-04-02

**Authors:** Shimaa Saber, Souham Meshoul, Khalid Amin, Paweł Pławiak, Mohamed Hammad

**Affiliations:** 1 Information Technology Department, Faculty of Computers and Information, Menoufia University, Shibin El Kom 32511, Egypt; shimaa.saber@ci.menofia.edu.eg (S.S.); k.amin@ci.menofia.edu.eg (K.A.); 2 Department of Information Technology, College of Computer and Information Sciences, Princess Nourah bint Abdulrahman University, P.O. Box 84428, Riyadh 11671, Saudi Arabia; sbmeshoul@pnu.edu.sa; 3 Department of Computer Science, Faculty of Computer Science and Telecommunications, Cracow University of Technology, Warszawska 24, 31-155 Krakow, Poland; 4 Institute of Theoretical and Applied Informatics, Polish Academy of Sciences, Bałtycka 5, 44-100 Gliwice, Poland

**Keywords:** ECA, deep learning, PAM, person re-identification, multi-attention

## Abstract

Person re-identification (Re-ID) is a method for identifying the same individual via several non-interfering cameras. Person Re-ID has been felicitously applied to an assortment of computer vision applications. Due to the emergence of deep learning algorithms, person Re-ID techniques, which often involve the attention module, have gained remarkable success. Moreover, people’s traits are mostly similar, which makes distinguishing between them complicated. This paper presents a novel approach for person Re-ID, by introducing a multi-part feature network, that combines the position attention module (PAM) and the efficient channel attention (ECA). The goal is to enhance the accuracy and robustness of person Re-ID methods through the use of attention mechanisms. The proposed multi-part feature network employs the PAM to extract robust and discriminative features by utilizing channel, spatial, and temporal context information. The PAM learns the spatial interdependencies of features and extracts a greater variety of contextual information from local elements, hence enhancing their capacity for representation. The ECA captures local cross-channel interaction and reduces the model’s complexity, while maintaining accuracy. Inclusive experiments were executed on three publicly available person Re-ID datasets: Market-1501, DukeMTMC, and CUHK-03. The outcomes reveal that the suggested method outperforms existing state-of-the-art methods, and the rank-1 accuracy can achieve 95.93%, 89.77%, and 73.21% in trials on the public datasets Market-1501, DukeMTMC-reID, and CUHK03, respectively, and can reach 96.41%, 94.08%, and 91.21% after re-ranking. The proposed method demonstrates a high generalization capability and improves both quantitative and qualitative performance. Finally, the proposed multi-part feature network, with the combination of PAM and ECA, offers a promising solution for person Re-ID, by combining the benefits of temporal, spatial, and channel information. The results of this study evidence the effectiveness and potential of the suggested method for person Re-ID in computer vision applications.

## 1. Introduction

**Figure 1:**
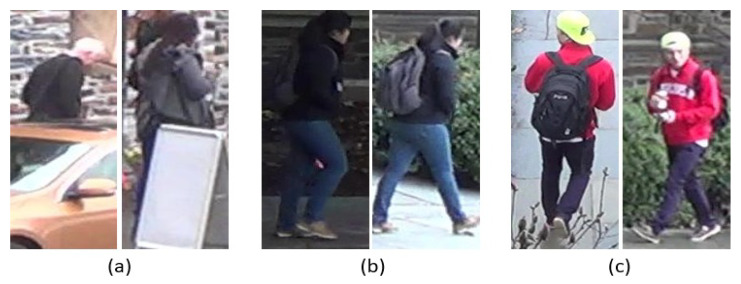
Some difficult issues within the DukeMTMC dataset. (**a**) Occlusions, (**b**) illumination differences, (**c**) pose variations.

Person re-identification (Re-ID) is one of the computer vision tasks that aims to match a target individual across many camera perspectives. It has become an increasingly significant field of research in recent years, particularly in the area of surveillance and security. The main motivation for person Re-ID is to enable effective tracking of individuals in complex and crowded environments, such as airports, train stations, and public places [1,2]. However, the mission of person Re-ID faces several challenges that make it difficult to achieve high levels of accuracy. These challenges include variations in lighting conditions, occlusions, changes in appearance, and perspective changes [3], as described in Figure 1. Additionally, person Re-ID is a large-scale and complex problem, as it requires searching through large databases of images to find the correct match.

Previous approaches to person Re-ID have included the use of hand-crafted features, metric learning algorithms, and deep learning [1,4,5]. The traditional methods contain hand-crafted feature extraction and distance metrics. Hand-crafted feature extraction is utilized to obtain more discriminative information from the image of a person, by using methods such as color histograms, texture features, scale-invariant feature transform (SIFT), local binary pattern (LBP), and other techniques. Metric learning algorithms have been used to match images using distance metrics, support vector machines (SVMs), neural networks (NN), cross-view quadratic discriminant analysis (XQDA), nearest neighbors (KNN), and other metric learning types [6,7], but this approach requires expert knowledge in feature design and is limited in its ability to capture complex relationships between images. Metric learning algorithms aim to learn a distance metric optimized for person Re-ID, but they still have limitations, such as difficulty learning an appropriate mapping for large-scale datasets. Deep learning, particularly convolutional neural networks (CNNs), has significantly improved the accuracy of person Re-ID algorithms, by learning a feature representation directly from the raw images. However, deep learning approaches also present new challenges, such as the need for large amounts of labeled data and the computational requirements of training large models. The best approach to person Re-ID depends on the specific requirements of the task, but deep learning has had a significant impact on the field.

CNNs have proven to be an efficacious tool for addressing the issue of person Re-ID. They are capable of learning and capturing the discriminative features of the input images, and can be learned from end-to-end on large datasets [5]. Additionally, CNNs can be fine-tuned for specific datasets, making it possible to improve their performance in challenging scenarios [8]. By leveraging the ability of CNNs to automatically learn and extract features, person Re-ID algorithms have achieved significant improvements in accuracy, making them an important tool for overcoming the perplexing problem of person Re-ID.

In the past decade, person Re-ID has attracted a great deal of interest, due to its utility in a range of computer vision applications, such as video surveillance and person tracking [6]. Re-ID attempts to identify a person of interest across numerous, non-overlapping cameras. Recently proposed methods in person Re-ID tasks show good performance while using the attention mechanism, by focusing on more relevant characteristics [2,6]. In addition, most Re-ID methods depend on global features, that focus on the overall information in the image of a person and ignore the spatial structure of that person, so recently, many Re-ID methods have mainly extracted local features for re-identification, to improve the extracted features [9,10].

Despite the success of person recognition methods, identifying the same person in different cameras remains a difficult task, particularly in scenarios where the features of a person repeatedly change. To tackle this challenge, we provide a new person Re-ID method, that combines attention learning with a pre-trained model, which is a deep CNN that has already been trained to find informative and strong features in images, making the Re-ID process much easier and faster compared to models that are learned from scratch. Our system employs an attention mechanism that combines the position attention module (PAM) and the efficient channel attention (ECA). The PAM captures spatial, temporal, and channel context information, which improves the representation capability of the local features. The ECA reduces the model’s complexity while maintaining accuracy, by capturing local cross-channel interactions.

Our contributions in this paper are twofold: (1) we introduce attention learning combined with a pre-trained model, for person Re-ID, which outperforms existing methods, and (2) we present an attention mechanism that combines the PAM and ECA, which improves the representation capability and decreases the complexity of the model, while preserving accuracy.

The remainder of the article is structured as follows: Section 2 covers relevant research in person Re-ID. Section 3 introduces the suggested method. Section 4 displays the research results. Section 5 shows the analysis study. Finally, Section 6 wraps up the paper and suggests future directions.

## 2. Related Work

In recent years, person Re-ID has become a crucial task in video observation, and has gained significant consideration in computer vision. Several approaches, including metric learning, hand-crafted features, and deep learning, have been proposed for this problem. In this part, we provide a summary of the most recent and relevant research in this area, with a focus on deep learning methods.

### 2.1. Hand-Crafted Feature-Based Person Re-ID

Manual feature extraction and metric learning design are person Re-ID’s traditional methods; they rely on detecting low-level appearance features from the requisite image characteristics, such as shapes, colors, and textures [11]. Support vector machines (SVMs), neural networks (NN), nearest neighbors (KNN), and others, are metric learning types that minimize the distance between traits of the same person. Feature descriptors and metric learning are two independent stages. Liao et al. [9] presented a method that incorporates effective feature detection with metric learning. They suggested local maximal occurrence (LOMO) as a traits descriptor, that represents the image by extracting the histogram for colors using the texture histogram and sliding window with scale-invariant local ternary mode. Also, they used cross-view quadratic discriminant analysis (XQDA) for matching between features. Yang et al. [11] presented a method for extracting the features dependent on colors, that are called salient color names-based color descriptors (SCNCD), and they used the KISSME technique for metric learning. SCNCD divides the image into six parts equally and then computes the histogram for different spaces of color on all parts, to make the definitive extracted features sensitive to changes in illumination.

### 2.2. Hybrid Feature-Based Person Re-ID

The hybrid method combines deep learning with metric learning. The authors extract the features by utilizing a convolutional neural network and metric learning for classification. Saber et al. [6] used VGG-Net as a person representation, which provides a deep learning mechanism for person identification, and they selected the most estimated layers, to gain a useful feature description for the person. Subsequently, for person matching, a support vector classifier (SVC) was used, which eliminated the issue of using a small dataset. Jayapriya et al. [10] used CNN to extract traits from sequential information. This strategy combined the prioritized chromatic texture image (PCTimg) with the original images, then entered them into the CNN to detect the traits. XQDA is employed for the classification. Wang et al. [12] developed a Siamese model, that employed XQDA to learn a discriminant metric, and extracted traits from deep networks to obtain spatiotemporal information about the person.

### 2.3. Deep Learned Feature-Based Person Re-ID

Deep learning is based on neural network algorithms and has become a prevalent offshoot of machine learning [13]. Deep learning algorithms employ multiple transformation layers with intricate constructions, in an effort to demonstrate high-level characteristics in data. In contrast to traditional methods, deep learning methods incorporate feature descriptions and similarity measures into an entire model. There are different kinds of architectures for deep learning-based methods, like attention-based methods and part-based methods.

Attention-based methods aim to carefully choose high-interest areas from input data, while disregarding other areas, with weak or no discriminative features. Attention modules concentrate on extracting regions with extremely distinguishing characteristics. Guodong et al. [14] proposed a hybrid architecture for CNN, that allows the network to concentrate on global and local discriminatory features for a person’s image. They introduced a method called feature mask network (FMN). Wei et al. [15] established the global–local-alignment descriptor (GLAD) network, that appreciates the skeletons and splits the image by using the deeper cut. GLAD is intended to detect both local traits from separated images and global traits from the whole body. Masked graph attention network (MGAT) is a network designed by Bao et al. [16], that concentrates on the relationship between individual images and their labels, while ignoring the global mutual information present in the full sample set. The MGAT is dependent on a plenary network that extracts features, where nodes can concentrate on the characteristics of others in a directly navigable mode in the form of a mask matrix, with label information for guiding.

Part-based Re-ID approaches, elicit image areas to discover distinctive part-level features, established on accurate part-level cues that are often neglected when retrieving global traits. Part-based convolutional baseline (PCB) network was suggested in [17], which uses uniform segmentation on the convolution layer to interpret part-level data, by dividing the entire body into six horizontally running stripes in the feature map. Each component feature vector is supplied to a classifier, which generates an ID-prediction loss, that is independent for each part. Tian et al. [18] proposed a joint learning network that focuses on learning more distinctive and powerful features. They applied a global branch to learn the most distinctive global-level traits, and they divided the extracted map of traits into N parts, which are taken as inputs into a distinctive network that comprehends the local-level features. Afterward, they generate a local loss by combining N-part losses. They can then obtain a desirable total loss by combining local and global losses. A Siamese multiple granularity network (SMGN), with two major branches, was proposed by Li et al. [19], for learning the local and global characteristics of a person independently. The retrieved features of the two branches are combined as multiple features for personal images, and multiple loss functions are employed to enhance their performance.

From the above discussion, it is seen that previous studies have tried to enhance the person Re-ID performance using different methods. However, most of these methods have limitations, and do not perform well on large datasets. Our proposed method overcomes these limitations, by combining attention learning with a pre-trained model, which outperforms the existing methods on large datasets. The main difference between the proposed work and the related work, is that the proposed method combines the PAM and ECA, to extract features from temporal, spatial, and channel contexts. This is a new approach that has not been explored in previous studies. The proposed multi-part feature network, with the combination of PAM and ECA, has great potential to solve the problem of person Re-ID successfully, as it combines the benefits of temporal, spatial, and channel information. To summarize, the proposed method differs from previous studies, in that it combines the PAM and ECA to extract features from multiple contexts, with a high potential to achieve better results than existing methods. Table 1 summarizes the main differences between the proposed work and related work in the field of person Re-ID.

## 3. Methodology

**Figure 2:**
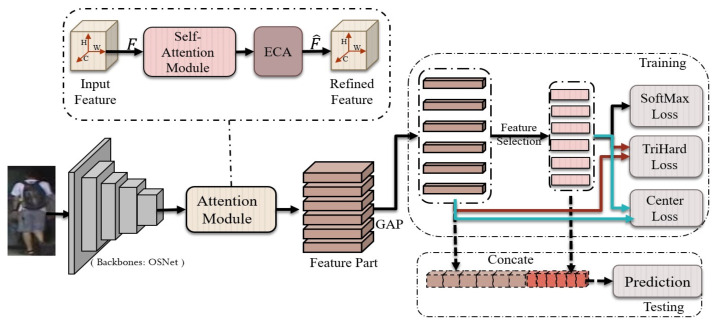
The architecture of the multi-part feature network.

In this section, we depict the overall structure of a multi-part feature network for a person Re-ID task, that can independently learn extensive information from different parts of features, and the features from these parts can be merged for prediction. Then, we describe the two attention modules that are utilized to reduce the impact of irrelevant background, while concentrating on discriminative features of a person’s appearance. Finally, we describe the loss functions that are utilized. OSNet [20] acts as the foundation for our network structure, as shown in Figure 2.

### 3.1. Baseline Configuration

We utilized OSNet [20] as a feature extractor for combining heterogeneous and homogeneous features, as well as a relatively lightweight network capable of developing performance, while avoiding over-fitting. OSNet [20] is built by stacking the bottleneck layer by layer, to decrease the parameter numbers, thereby lowering the computational cost.

### 3.2. Position Attention Module

In the person scenario, we observed that distinctive trait representations are fundamental for person Re-ID, which may be achieved by understanding contextual information. To extract contextual information from local traits, we utilized a position attention module (PAM), which extracts much information derived from local characteristics, thereby improving their ability to represent the features.

**Figure 3:**
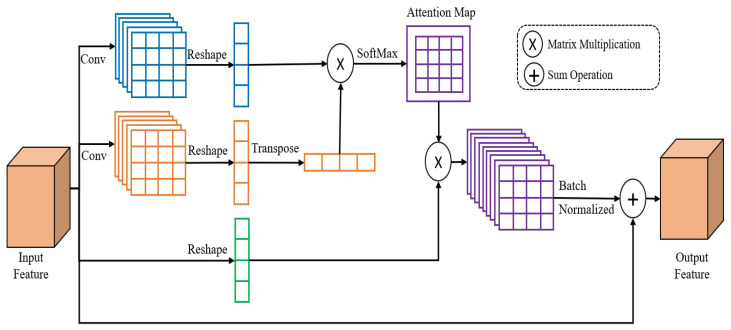
PAM attention module.

The structure of the position attention module (PAM) [21], which is made for detecting and collecting the relevant pixels in the spatial domain, is depicted in Figure 3. The feature $F\in R^{C\times H\times W}$, where *C* is the number of channels, *H* is the spatial dimension height, and *W* is the spatial dimension width of an input tensor. We first feed the feature maps in the first branches into a convolution layer, to produce the new feature maps $F1\in R^{C/16\times H\times W}$, then we reshape F1 to $R^{C/16\times N}$, where *N* is the number of the pixels, which is equal to H×W. To obtain F2 for the second branch, we apply the same mechanism as for the first branch. Following that, we multiply the transpositions of F2 and F1 using matrix multiplication, and then utilize a softmax layer to compute the attention map $S\in R^{N\times N}$. Then, we execute matrix multiplication between S and the reshaping of the input feature, to get the feature to $R\in^{C\times H\times W}$. Ultimately, the definitive output $\in R^{C\times H\times W}$ is obtained by applying the batch normalization and then executing an operation of element-wise sum with the input features. Generally, in the original PAM, the third branch began with the 2D convolution layer, and we removed this layer to decrease the training time and increase the accuracy of our Re-ID method.

### 3.3. Efficient Channel Attention (ECA)

The channel attention module has shown significant potency to enhance the effectiveness of deep CNN. Channel attention is utilized to ameliorate the features of different channels, by simulating the significance of all channels in the feature. One of these channel attention modules is efficient channel attention (ECA). ECA detects interactions on the local cross-channel, by analyzing the channel and its neighbors. ECA minimizes the parameter numbers and reduces the model’s complexity, while maintaining precision.

**Figure 4:**
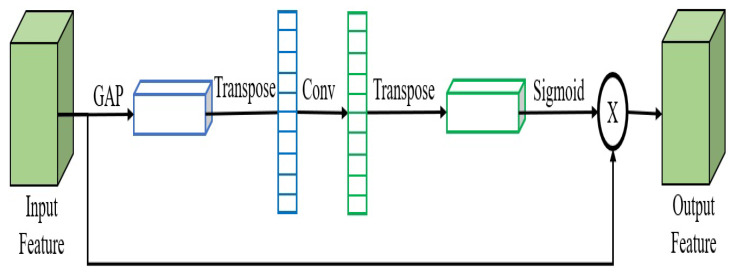
ECA attention module.

ECA’s structure was proposed in [22]. To begin, as illustrated in Figure 4, a global average pooling (GAP) method is used, to reduce the size dimension of the input feature. After that, the weights of the channel are derived by a 1D convolution with a kernel size of three. Lastly, a sigmoid function is used, to obtain the final attention weights. Channels’ local interactive information can be reserved in this manner.

### 3.4. Loss Functions

As the final description for the person Re-ID features, we concatenate the feature vectors from the GAP and feature selection. Our loss function is gathered from ID loss (softmax loss) [23] for the six parts of the selected feature, and from a hard-margin triplet loss [24] and a center loss [25], for the concatenated feature. As demonstrated in Figure 2, each classifier forecasts the identification of the input image, namely,

(1) $$L_{total}=\sum L_{id}+\beta \times L_{H-triplet}+\alpha \times L_{center}$$

where β and α are weighting factors.

The cross-entropy loss (softmax loss) for the learned features, $f_{i}$, with label smoothing [23], is given as:

(2) $$L_{id}=-\sum_{i=1}^{N}\times q_{yi}\times log\frac{e^{\left(w_{i}\times f_{i}+b_{i}\right)}}{\sum_{j=1}^{C}e^{\left(w_{j}\times f_{j}+b_{j}\right)}}$$

where *N* is the batch size, *C* is the identity class number, $f_{i}$ is the extracted feature, $w_{i}$ and $b_{i}$ are the weighted and bias for class *i*, respectively, and $q_{yi}$ is the ground truth of the labels.

By obtaining many centers for all identity classes, hard triplet loss [24] outperforms softmax loss. However, the max function is required, to find the closest center for each identity class, and it is not smooth, thus the function can be sensitive between several centers. Smoothing of the max function in the softmax loss, can be utilized to enhance robustness. The hard triplet loss for the learned feature $f_{i}$, is given as:

(3) $$L_{H-triplet}=-\sum_{i=1}^{N}log\frac{e^{\lambda (S_{i,y_{i}}-\delta )}}{e^{\lambda (S_{i,y_{i}}-\delta )}+\sum_{j\neq y_{i}^{e^{\lambda S_{i,j}}}}}$$

where λ is compensated to optimize a smoothed triplet loss, δ is a predefined margin, and $S_{\left(i,j\right)}$ is the similarity between feature $f_{i}$ and the class *j*.

The center loss [25] is used to decrease intra-class variance between each sample in the mini-batch, while maintaining the features of the various classes separately. It can also reduce the distance within the class, so the compression of the samples within the class can be realized. The center loss function is written as follows:

(4) $$L_{center}=\frac{1}{2}\sum_{i-1}^{N}{∥f_{i}-C_{y_{i}}∥}_{2}^{2}$$

where $f_{i}$ is the detected feature, and $C_{y_{i}}$ is the updated deep feature.

## 4. Experimental Results and Discussion

In this section, we will carry out comprehensive experiments to confirm the viability of the suggested procedure. This section is arranged as follows: 1. provides three common datasets; 2. explains the specifics of implementation; 3. elucidates the protocols employed to test our strategy; and 4. compares the introduced approach to competing approaches on the relevant datasets.

### 4.1. The Utilized Datasets

To evolve and test the introduced model, we employed three diverse common datasets, as shown in Table 2, which are the fundamental datasets employed for the person Re-ID task.

CUHK03 [26]: was the first considerable dataset for a person Re-ID task. Images in this dataset contain the person detected by manual labeling and deformable part models (DPM). It contains 1467 identities, captured by two non-overlapping cameras.

Market-1501 [27]: was gathered by six separate cameras, at Tsinghua University. It contains 1501 identities, and images in this dataset contain the person detected by manual labeling and deformable part models (DPM), it also has 2793 false images because of the DPM detector.

DukeMTMC [7]: is one of the large-scale datasets. Eight cameras were utilized in the DukeMTMC dataset, to track multiple targets. It contains 1812 persons, and the person in each image is manually labeled.

### 4.2. Specifics of Implementation

Our introduced network was tested on a PC that uses NVIDIA RTX3060 12GB. OSNet [20] was pre-trained on ImageNet [28], where we omitted the GAP layer and fully connected layers. To be more specific, all image sizes were changed to 384 × 128 before being entered into the network. For training, our proposed network extracted features, and the optimizer of the network was the stochastic gradient descent (SGD) algorithm, with a learning rate of 0.04, decay rate of 0.1, and momentum of 0.9. In the training set, CUHK03 has 767 individuals and 7368 photos, while in the testing set, there are an additional 700 individuals and 6732 images. Market-1501 has 751 persons in the training set, with 12,936 images, and another 750 persons in the testing set, with 16,482 images. DukeMTMC has 702 identities, with 16,522 images in the training set, and another 702 identities in the testing set, with 16,426 images.

### 4.3. Metric Protocol

We employed the single-shot approach in our experiment, which allows a thorough comparison. The cumulative matching characteristic (CMC) [29] and mean average precision (mAP) [30] were utilized to evaluate the person Re-ID performance. To improve performance even further, we added the re-ranking method [31], dependent on k-reciprocal encoding, to our method. The re-ranking operation was utilized in the testing phase.

### 4.4. Evaluation on the Used Datasets

The introduced method appears to have excellent results compared to the preceding methods. Prior to discussing its accuracy on the three datasets, the introduced approach is evaluated against the state-of-the-art methods as follows:

**Market-1501 database**: Table 3 shows the competitive fineness results for the proposed technique and other person Re-ID methods, using the Market-1501 dataset. Our proposed method affords enhanced outcomes compared to the other methods. The introduced method achieves 95.93%, compared to the highest score achieved by DM-OSNet [8], of 95.61%. However, DRL-Net [32] achieves the highest mean average precision (mAP) score, of 89.9%, while the proposed method achieves a score of 87.57%. By utilizing the re-ranking [31], the proposed method achieves even higher results, with a rank-1 accuracy of 96.41% and an mAP of 94.15%. The results demonstrate that the proposed method performs well compared to other state-of-the-art techniques. However, there are still some limitations, as some methods perform better in certain aspects, such as DRL-Net for mAP. The findings of this study could have important implications for the development of more accurate person re-identification systems in real-world applications.

**DukeMTMC-reID database:** This has more challenges than in the Market-1501 dataset, due to the greater number of camera views and noisy backgrounds, that gives more variation within classes. Table 3 also presents the results of various person re-identification methods on the DukeMTMC dataset, including our proposed method, with and without re-ranking. In terms of rank-1 accuracy, our introduced method achieves 89.77%, compared to the highest score achieved by AET-Net [33], of 89.5%. However, AET-Net [33] achieves the highest mean average precision (mAP) score, 80.1%, while the proposed method achieves a score of 78.62%. Using the re-ranking technique, our proposed method improved its performance to 94.08% and 92.22%, in rank-1 and mAP, respectively.

**CUHK03 database:** Table 4 presents the results of various person re-identification methods on the CUHK03 dataset, including our proposed method, with and without re-ranking. Our proposed method achieves impressive performance on both labeled and detected types, outperforming other methods by 3.01% and 2.65%, respectively. Moreover, the use of the re-ranking technique results in a substantial improvement in performance, with an increase of 18% for labeled and 17.51% for detected types. These results demonstrate the effectiveness of our proposed method and highlight its potential to improve on state-of-the-art person re-identification methods. When comparing our proposed method to other state-of-the-art methods, it is clear that our approach presents several strengths. For instance, our method outperforms the widely used PCB [17] method by a significant margin, achieving an improvement of 10.55% in rank-1 accuracy for detected types. Additionally, our proposed method outperforms the HAN [34] method by 26.71% in mAP, for labeled types. However, our method does have some limitations, such as being computationally expensive, due to the high-dimensional feature extraction required. Despite these limitations, our proposed method demonstrates superior performance, and the results indicate that it has the potential to be a useful tool for person re-identification in real-world scenarios.

**Table 3:** Network technical change comparison on the Market-1501 dataset.

Method	Market-1501		DukeMTMC-reID	
	Rank-1	mAP	Rank-1	mAP
PCB [17]	92.3	77.4	81.8	66.1
RJLN [18]	93.7	81.9	85.5	73.1
PIE [35]	87.33	69.25	80.84	64.09
AlignedReID++ [36]	91.8	79.1	82.1	69.7
PGFA [37]	91.2	76.8	82.6	65.5
Deep Person [38]	92.31	79.58	80.90	64.80
FMN [14]	85.99	67.12	74.51	56.88
FMN+re-rank [14]	87.92	80.62	79.52	72.79
FPO [39]	91.81	79.23	81.0	78.0
DCNN [40]	90.2	82.7	80.6	64.1
HAN [34]	91.6	76.7	80.7	65.5
UANet [41]	91.3	76.5	82.1	65.2
UnityStyle [42]	91.8	76.5	80.38	64.32
SMGN [19]	94.1	79.2	86.1	75.3
SMGN + re-rank [19]	95.5	80.3	87.1	76.0
GCN [43]	88.65	74.15	-	-
ARFM [44]	88.02	76.13	81.53	65.94
AL-APR [45]	89.01	74.38	80.52	63.67
DUNet [46]	91.6	75.90	82.1	66.5
NFML [47]	95.3	86.4	89.2	76.2
EDAGAN [48]	85.36	64.52	74.19	51.90
CooRL [49]	89.5	74.3	78.9	65.2
Tri-GCN [2]	92.98	80.5	83.23	66.8
HOB-net [50]	94.7	86.3	88.2	77.2
SFBM [51]	95.3	85.4	88.6	74.5
VACNet [33]	95.1	86.1	89.5	77.1
twinsReID [52]	93.7	85.4	88.6	78.2
DM-OSNet [8]	95.61	87.36	89.18	78.26
TAFN [53]	94.7	86.2	85.9	74.8
MS-LS-RK [54]	92.3	88.3	86.5	81.7
AM0BH [55]	94.6	85.9	89.2	76.7
RANGEv2 [56]	94.7	86.8	87.0	78.2
DRL-Net [32]	94.7	89.9	88.1	76.6
AET-Net [33]	94.8	87.5	89.5	80.1
Our method	**95.93**	87.57	**89.77**	78.62
Our method+re-rank	**96.41**	**94.15**	**94.08**	**92.22**

**Table 4:** Network technical change comparison on the CUHK03 dataset (labeled and detected).

Method	CUHK03			
	Labeled		Detected	
	Rank-1	mAP	Rank-1	mAP
PCB [17]	-	-	61.3	54.2
PIE [35]	-	-	45.88	41.21
RJLN [18]	-	-	66.6	60.9
FMN [14]	41.0	38.1	42.6	39.2
FMN+re-rank [14]	46.0	47.6	47.5	48.5
HAN [34]	46.5	46.1	47.5	45.5
FPO [39]	65.60	60.16	63.07	56.31
UANet [41]	62.6	57.7	58.9	52.6
DUNet [46]	54.6	52.2	51.6	49.9
Tri-GCN [2]	68.29	61.59	65.86	58.21
HOB-net [50]	70.2	67.5	69.2	66.8
RANGEv2 [56]	64.3	67.4	61.6	64.6
Our method	**73.21**	67.34	**71.85**	66.16
Our method+re-rank	**91.21**	**91.4**	**89.36**	**89.29**

## 5. Research Analysis

In this section, we analyze the parameters of the Market-1501 dataset, including the effect of image size, the number of image parts, batch size, loss type, attention module type, and epoch number.

### 5.1. Comparison of Loss Function Change

In the training stage, our loss function gathers from cross-entropy, the hard triplet loss, and the center loss. To inspect the effect of the loss function, we performed experiments in which we performed cross-entropy loss with the triplet loss, center loss, or a combination of them, to confirm the efficacy of employing multiple losses. Table 5 showcases the performance of the proposed multiple loss function combinations on the three different datasets—Market-1501, DukeMTMC, and CUHK03 (both labeled and detected). As seen in the experimental results, utilizing many losses causes the network to exhibit varying degrees of accuracy enhancement on the three datasets, when compared to using only the softmax loss. For instance, using the combination of losses, outperformed the competition by 0.99% and 1.11% in rank-1 and mAP, respectively, on the DukeMTMC dataset. Similarly, the CUHK03 dataset increased by 1.14% and 1.64%, for labeled and detected sets, respectively, using the proposed method. In combination, the loss functions are fused, making them interactive, resulting in improved performance at the cost of speed, and the network converges towards greater performance. Generally, the results demonstrate that the proposed method is effective in enhancing the accuracy of the network and has the potential to improve state-of-the-art person re-identification.

### 5.2. Comparison of Attention Change

Many cutting-edge methodologies for person Re-ID tasks, make use of attention modules. To extract global features, we added the attention module, which consists of PAM and ECA, into the network. To investigate the efficiency of the suggested attention module in our framework, we conducted experiments on the Market-1501 dataset. Six structures are compared: only the network without the attention, the network with only one attention module (PAM or ECA), the network with changing the order of the attention modules (PAM after or before ECA), the network with the average of the attention modules (PAM and ECA), and the complete network. Table 6 shows the experimental results for the Market-1501 dataset, comparing the use of attention mechanisms with different configurations, against a baseline without attention mechanisms. The configurations of the attention module are denoted by the numbers in parentheses. As seen in the table, the use of attention mechanisms improves the network’s performance, achieving higher rank-1 and mAP scores compared to the baseline. Specifically, the best performing configuration is (1) (2), which utilizes both PAM and ECA attention mechanisms, achieving a rank-1 score of 95.93% and mAP of 87.57%, which represents a significant improvement, of 2.42% and 6.92%, respectively, compared to the baseline. Additionally, the results show that the PAM attention mechanism contributes more to the improvement than the ECA attention mechanism. Configuration (1)—which uses only PAM—achieved a higher rank-1 score than configuration (2) (1), which uses only ECA. This suggests that PAM is more effective in capturing long-range dependencies between features. The experimental results demonstrate the effectiveness of using attention mechanisms in improving the performance of person re-identification networks. The use of PAM and ECA attention mechanisms with appropriate configurations, can significantly improve the rank-1 and mAP scores, which are important performance metrics for person re-identification systems.

### 5.3. Comparison of Using Different Pre-Trained Models

To investigate the usefulness of the baseline that we chose, we compare the results of several baselines on different datasets. Our baselines for comparison are various versions of OSNet [20] and VGG16. Table 7 presents the experimental results obtained for the Market-1501, DukeMTMC, CUHK03-labeled, and CUHK03-detected datasets. Each row of the table corresponds to a different baseline network, while each column shows the rank-1 and mAP scores for a specific dataset. Our results demonstrate that the addition of suggested branches improves the performance of all baseline networks, with the most significant gains observed in the $OSNet_{X_{1}}$ network. In particular, our method achieved a rank-1 accuracy of 95.93% and a mAP score of 87.57% on the Market-1501 dataset, outperforming all other baseline networks. Our results also show that the VGG16 baseline network performed relatively poorly, with a rank-1 accuracy of only 90.25% and a mAP score of 74.86%. When comparing the results of each baseline network to the proposed method, it is evident that our method outperformed all baseline networks on all datasets, except for DukeMTMC, where $OSNet_{X_{1}}$, with our suggested addition, achieved the best performance. These results highlight the effectiveness of our proposed method in enhancing the performance of existing baseline networks. Furthermore, we observed some interesting trends and patterns in our data. For example, we found that $OSNet_{X_{1}}$ performed significantly better than other OSNet baselines after adding all the branches, except for rank-1 for DukeMTMC. Additionally, the $OSNet_{X_{0.75}}$ and $OSNet_{X_{1}}$ networks achieved higher performance than $OSNet_{X_{0.5}}$ and $OSNet_{X_{0.25}}$, respectively, suggesting that larger networks may better capture complex features in person re-identification. Overall, our study provides valuable insights into the effectiveness of our proposed method and the relative performance of different baseline networks in person re-identification.

### 5.4. Comparison of Network Architectural Change

To further interpret the results presented in Table 8, we can observe that the introduced strategy for applying the attention module after layer 4, provided the best performance in terms of rank-1, rank-5, rank-10, rank-20, and mAP scores. This indicates that the retrieved feature should include both coarse and fine information for a person’s representation, to make the attention module more successful. The results also show a clear trend of increasing performance with deeper layers, as adding the attention module after layers 3 and 4 improves performance, compared to adding it after layer 2. Moreover, the rank-1 score of 95.93%, achieved by applying the attention module after layer 4, is particularly noteworthy, as it represents a significant improvement over the other positions tested. These results demonstrate the effectiveness of the proposed strategy for integrating attention mechanisms into person re-identification models and suggest that future work in this area should explore the use of attention modules in conjunction with deeper network architectures.

### 5.5. Comparison of Image Size Change

To better understand the impact of image size on the performance of the proposed method, we conducted experiments using different image sizes, and evaluated the results in terms of rank-1, rank-5, rank-10, rank-20, and mAP scores, as shown in Table 9. It can be seen, that resizing the image to 384 × 128 provided the best performance in terms of rank-1 accuracy, with a score of 95.93%. The other image sizes had rank-1 scores ranging from 95.24% to 95.86%. This suggests that a larger image size can capture more detailed information about the person’s appearance, leading to better recognition performance. It is noteworthy that the choice of image size can also impact the overall computational cost of the system, and this factor should be considered when selecting the optimal image size for a given application.

### 5.6. Comparison of Feature Part Number

**Figure 5:**
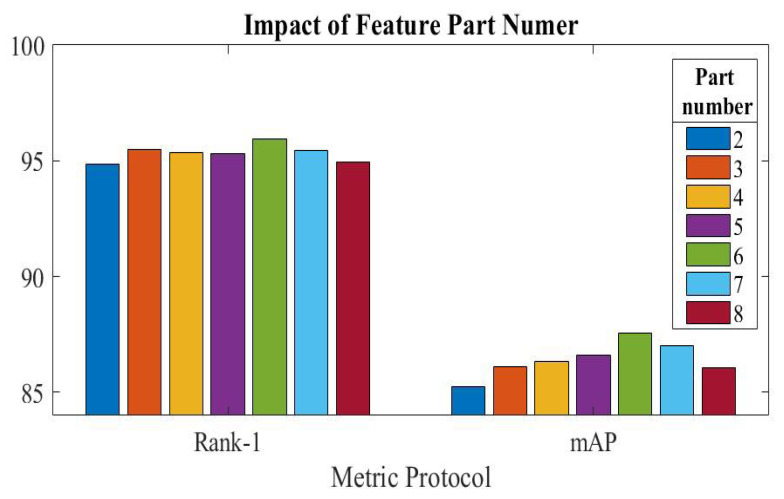
Performance of our proposed models under different feature part numbers.

We explored the impact of the part number of feature selection on overall Re-ID performance and tested it using the Market-1501 dataset. We attempted to train the provided model with a varied number of feature selection components. The output feature is a global feature if the part number is set to 1. Having six parts exhibits the best performance on the Market-1501 dataset, according to the results presented in Figure 5. The Re-ID performance begins to fall with adding further parts, indicating that too many components load the model training and therefore lower performance.

### 5.7. Comparison of Batch Size Change

**Figure 6:**
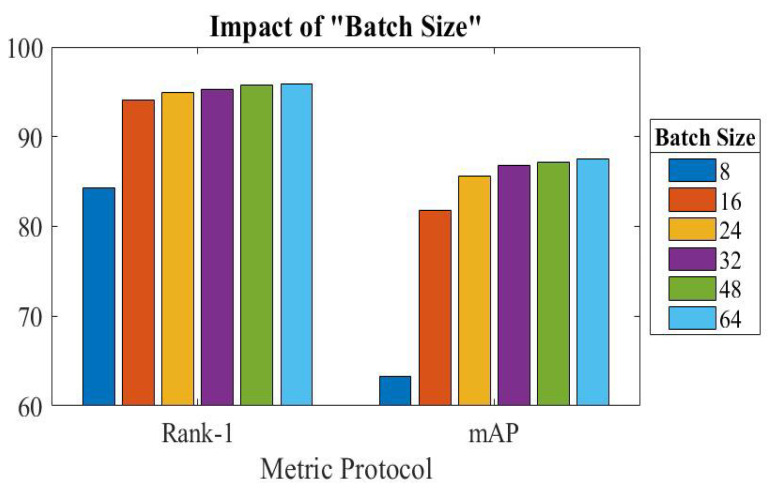
Effect of changing size of batch on Market-1501.

Here, we examined the effects of modifying the batch size in the training stage, where the batch size represents the number of images fed into the network. To examine the impacts of various batch sizes on the efficiency of our introduced network, comparative experiments were conducted. The largest batch size that could be used was 64, because of GPU limitations. Figure 6 illustrates the results of the experiment. As seen, performance changes as the batch size changes. The accuracy of the Market-1501 dataset may reach its highest value when the batch size is 64. Comparing the improvement to a batch size of 48, it is slight. As a result, performance varies by altering the batch size, and accuracy will continue to improve. We draw the conclusion that the processing of the samples’ derived features can be helped by increasing the batch size.

### 5.8. Impact of Numbers of Epoch

**Figure 7:**
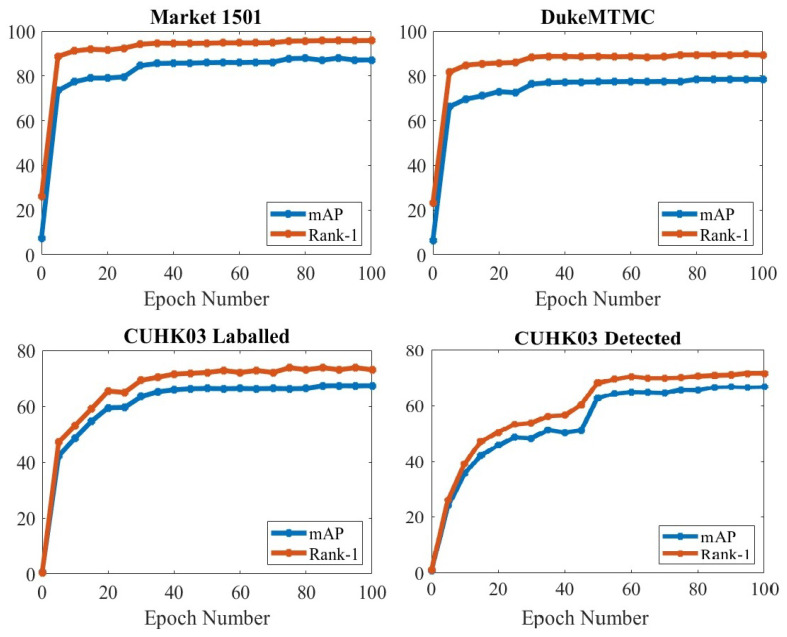
Effect of different numbers of epochs on the Market-1501, DukeMTMC, and CUHK03 (labeled and detected) datasets.

The empirical results for our introduced network during the training stage are illustrated in Figure 7, to test the effect of changing the number of epochs. Three different datasets were utilized in the experiment. This experiment comprised 100 training epochs and was evaluated every 5 training epochs. As illustrated in Figure 7, both rank-1 and mAP performance improve by increasing epoch numbers in the training stage, but the difference is slight until epoch 35 in the Market-1501, DukeMTMC, and CUHK03-labeled datasets, unlike the CUHK03-detected dataset, which needs to reach epoch 55 before the change becomes small.

## 6. Conclusions

This research presents a multi-part feature network for individual Re-ID, which combines the position attention module with efficient channel attention, to improve the robustness and discrimination of the features. The suggested attention mechanism utilizes temporal, spatial, and channel context information, to extract a broader variety of contextual information from local features, hence enhancing their capacity for representation. Under the restrictions of numerous losses, the methods we propose can produce resilient feature representations. Extensive testing on three datasets revealed that the proposed strategy outperformed state-of-the-art techniques and was highly generalizable. The results indicate that the suggested strategy enhances both quantitative and qualitative methods for re-identifying individuals. In the future, we intend to investigate and expand the introduced method, to improve the precision and efficacy of person Re-ID. 

## Figures and Tables

**Table 1:** Summary of the main differences between the proposed work and related work.

Approach	Main Focus	Techniques Used
Hand-crafted	Detecting low-level appearance features	SVM, NN, KNN, feature descriptors, metric learning
Hybrid	Combining deep learning with metric learning	CNN, metric learning, support vector classifiers
Deep learned	Using deep learning algorithms	Attention-based, part-based methods, CNN
Proposed	Multi-part, enhancing accuracy and robustness	Position attention module, efficient channel attention, multi-part feature network

**Table 2:** Description of the employed datasets in this research.

Dataset	Year	Camera	Person	Images	Challenges
CUHK03	2014	2	1467	13,164	Alignment variation, occlusion, missing body parts
Market-1501	2015	6	1501	32,217	Variations in scale, illumination, and pose, occlusion, background noise, alignment variation, occlusion, missing body parts
DukeMTMC	2017	8	1812	36,441	

**Table 5:** Performance of Re-ID models under different loss functions (× loss not employed, 🗸 loss employed).

Loss			Market-1501		DukeMTMC		CUHK03			
							Labeled		Detected	
**Cross-Entropy**	**Tri-Hard**	**Center**	**Rank-1**	**mAP**	**Rank-1**	**mAP**	**Rank-1**	**mAP**	**Rank-1**	**mAP**
🗸	×	×	94.86	86.17	88.78	77.6	72.07	66.23	70.21	64.92
🗸	×	🗸	95.01	86.32	89.00	77.79	72.25	66.41	70.34	65.20
🗸	🗸	×	95.74	87.12	89.53	78.21	72.94	66.91	71.66	65.79
🗸	🗸	🗸	95.93	87.57	89.77	78.62	73.21	67.34	71.85	66.16

**Table 6:** Quantitative comparison of the attention module type on the Market-1501 dataset (where the number is the order of the attention modules).

Attention		Market-1501	
PAM	ECA	Rank-1	mAP
-	-	93.04	80.04
(1)	-	95.81	86.97
-	(1)	93.13	80.11
(1)	(2)	95.93	87.57
(2)	(1)	95.54	86.3
(1)	(1)	95.46	86.96

**Table 7:** Performance of proposed strategy of RE-ID under different baselines.

Baseline	Market-1501		DukeMTMC		CUHK03-Labeled		CUHK03-Detected	
	Rank-1	mAP	Rank-1	mAP	Rank-1	mAP	Rank-1	mAP
VGG16	90.25	74.86	74.92	55.47	57.21	51.44	51.07	45.39
OSNet_IBN_X_1	95.00	84.54	89.06	76.54	68.36	63.62	65.79	60.23
OSNet _X_0.25	88.8	72.61	81.83	64.14	55.71	49.99	51.64	46.90
OSNet _X_0.5	93.4	82.64	87.98	73.19	69.5	63.06	64.29	58.17
OSNet _X_0.75	95.42	86.18	89.96	77.2	72.93	67.35	68.57	62.92
OSNet _X_1	95.93	87.57	89.77	78.62	73.21	67.34	71.85	66.16

**Table 8:** Comparison of performance when changing attention position.

Position	Market-1501				
	Rank-1	Rank-5	Rank-10	Rank-20	mAP
Layer 2	87.55	94.71	96.17	97.95	68.6
Layer 3	93.72	97.39	98.52	99.17	82.17
Layer 3 and 4	94.2	97.45	98.4	99.05	82.12
Layer 4	95.93	98.13	99.02	99.44	87.57

**Table 9:** Performance of Re-ID models under changing image sizes.

Image Size		Market-1501				
Height	Width	Rank-1	Rank-5	Rank-10	Rank-20	mAP
256	128	95.51	98.01	99.02	99.32	86.58
	192	95.24	98.31	98.9	99.35	86.12
320	128	95.25	98.13	98.96	99.32	87.97
	192	95.6	98.4	99.05	99.38	86.55
384	128	95.93	98.13	99.02	99.44	87.57
	192	95.86	98.31	99.05	99.44	87.15

## Data Availability

Not applicable.
